# Causal relationships between breast cancer risk factors based on mammographic features

**DOI:** 10.1186/s13058-023-01733-1

**Published:** 2023-10-25

**Authors:** Zhoufeng Ye, Tuong L. Nguyen, Gillian S. Dite, Robert J. MacInnis, Daniel F. Schmidt, Enes Makalic, Osamah M. Al-Qershi, Minh Bui, Vivienne F. C. Esser, James G. Dowty, Ho N. Trinh, Christopher F. Evans, Maxine Tan, Joohon Sung, Mark A. Jenkins, Graham G. Giles, Melissa C. Southey, John L. Hopper, Shuai Li

**Affiliations:** 1 Centre for Epidemiology and Biostatistics, Melbourne School of Population and Global Health, The University of Melbourne, Parkville, VIC 3051 Australia; 2 Genetic Technologies Limited, Fitzroy, VIC 3065 Australia; 3 Cancer Epidemiology Division, Cancer Council Victoria, Melbourne, VIC 3004 Australia; 4 Department of Data Science and AI, Faculty of IT, Monash University, Melbourne, Australia; 5 Electrical and Computer Systems Engineering Discipline, School of Engineering, Monash University Malaysia, 47500 Sunway City, Malaysia; 6 School of Electrical and Computer Engineering, The University of Oklahoma, Norman, OK 73019 USA; 7 Department of Public Health Sciences, Division of Genome and Health Big Data, Graduate School of Public Health, Seoul National University, Seoul, 08826 Korea; 8 Precision Medicine, School of Clinical Sciences at Monash Health, Monash University, Clayton, VIC 3168 Australia; 9 Department of Clinical Pathology, The University of Melbourne, Parkville, VIC 3051 Australia; 10 Department of Public Health and Primary Care, Centre for Cancer Genetic Epidemiology, University of Cambridge, Cambridge, CB1 8RN UK; 11 Murdoch Children’s Research Institute, Royal Children’s Hospital, Parkville, VIC 3051 Australia

**Keywords:** Breast cancer, Mammographic density, Textural feature, ICE FALCON, Causal inference

## Abstract

**Background:**

Mammogram risk scores based on texture and density defined by different brightness thresholds are associated with breast cancer risk differently and could reveal distinct information about breast cancer risk. We aimed to investigate causal relationships between these intercorrelated mammogram risk scores to determine their relevance to breast cancer aetiology.

**Methods:**

We used digitised mammograms for 371 monozygotic twin pairs, aged 40–70 years without a prior diagnosis of breast cancer at the time of mammography, from the Australian Mammographic Density Twins and Sisters Study. We generated normalised, age-adjusted, and standardised risk scores based on textures using the Cirrus algorithm and on three spatially independent dense areas defined by increasing brightness threshold: light areas, bright areas, and brightest areas. Causal inference was made using the Inference about Causation from Examination of FAmilial CONfounding (ICE FALCON) method.

**Results:**

The mammogram risk scores were correlated within twin pairs and with each other (*r* = 0.22–0.81; all *P* < 0.005). We estimated that 28–92% of the associations between the risk scores could be attributed to causal relationships between the scores, with the rest attributed to familial confounders shared by the scores. There was consistent evidence for positive causal effects: of Cirrus, light areas, and bright areas on the brightest areas (accounting for 34%, 55%, and 85% of the associations, respectively); and of light areas and bright areas on Cirrus (accounting for 37% and 28%, respectively).

**Conclusions:**

In a mammogram, the lighter (less dense) areas have a causal effect on the brightest (highly dense) areas, including through a causal pathway via textural features. These causal relationships help us gain insight into the relative aetiological importance of different mammographic features in breast cancer. For example our findings are consistent with the brightest areas being more aetiologically important than lighter areas for screen-detected breast cancer; conversely, light areas being more aetiologically important for interval breast cancer. Additionally, specific textural features capture aetiologically independent breast cancer risk information from dense areas. These findings highlight the utility of ICE FALCON and family data in decomposing the associations between intercorrelated disease biomarkers into distinct biological pathways.

**Supplementary Information:**

The online version contains supplementary material available at 10.1186/s13058-023-01733-1.

## Introduction

Mammographic density refers to the areas of a two-dimensional mammogram appears to be white. This is conventionally defined based on pixel threshold that can differentiate light or bright regions from dark regions, and can be measured using the semi-automated CUMULUS software, developed by Yaffe et al. in the 1990s [1]. We call this measure Cumulus. After transforming to normality and adjusting for age and body mass index (BMI), Cumulus measure is an established risk factor for breast cancer [2].

**Fig. 1:**
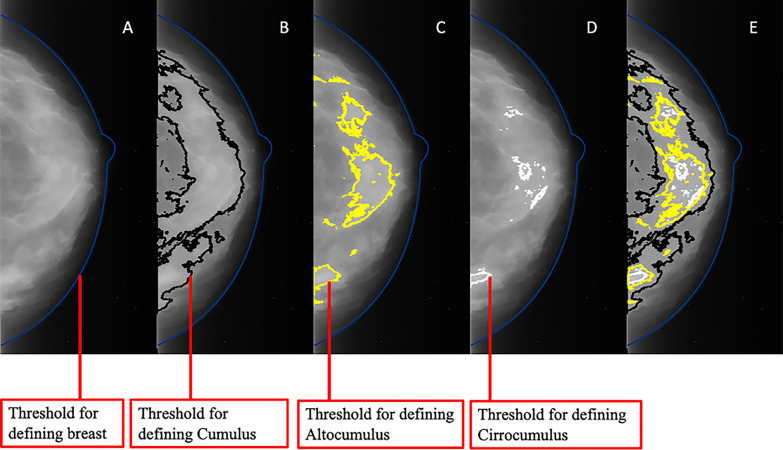
Example of mammographic areas divided into three areas defined by the breast area and density at increasing pixel intensity thresholds, using the same image. **A**: reference image; **B**: Cumulus; **C**: Altocumulus; **D**: Cirrocumulus; and **E**: All thresholds combined. The light areas are the Cumulus regions subtracting Altocumulus regions. The bright areas are the Altocumulus regions subtracting Cirrocumulus regions. The brightest areas are the Cirrocumulus regions

We found that two additional mammographic density measures defined by successively higher pixel brightness thresholds and called Altocumulus and Cirrocumulus, respectively (Fig. 1), when similarly transformed and adjusted can better predict risk of screen-detected breast cancer [3]. Moreover, when fitted together, the breast cancer risk association with Cumulus measure was attenuated far greater than the associations with Altocumulus and Cirrocumulus. Interval breast cancer risk, however, was better predicted by Cumulus [4, 5]. It is important to note that the dense areas measured by these three measures overlap with each other, specifically with Cirrocumulus being contained within Altocumulus, which, in turn, is encompassed by Cumulus (Fig. 1). Therefore, the mammographic image components defined by increasing pixel brightness threshold have the potential to reveal different information about breast cancer risk. This is also supported by recent findings from the Women's Environment, Cancer, and Radiation Epidemiology (WECARE) study that the dense areas defined by Cirrocumulus outperform another two dense areas, i.e. the dense areas between the threshold of Cirrocumulus and the threshold of Altocumulus, and between the threshold of Altocumulus and the threshold of Cumulus, respectively, in terms of predicting contralateral breast cancer risk [6]; see the details in “Discussion” section.

As well as mammographic density, the breast parenchyma could possess other mammographic patterns, such as textures, that predict breast cancer risk [7]. For example we developed an automated textural feature-based mammogram risk score, called Cirrus [8]. We found that Cirrus can improve risk prediction for interval cancers when fitted with Cumulus, as well as improve risk prediction for screen-detected and young-age-at-diagnosis breast cancer when fitted with Cirrocumulus [9, 10]. The associations between Cirrus and these three types of breast cancer remained after fitting the density measures. It is, therefore, possible that Cirrus contains independent and intrinsic risk information about breast cancer risk, especially given that pixel counting above a certain brightness level was not used as a criterion in its creation.

The different mammogram risk scores above are correlated with one another to varying degrees [4, 5, 10, 11]. One potential explanation is the spatial overlap of the density measures. Other studies have also identified associations between Cumulus and textural features [7, 12, 13] but the reasons for this have yet to be addressed.

We have consistently found that when the different mammogram risk scores are fitted together, their breast cancer risk gradients do not necessarily attenuate towards the null to the same extent as would be expected if their associations with one another were due solely to confounding [9, 10]. In particular, for screen-detected and young-age-at-diagnosis breast cancer, the Cumulus association became almost null after being fitted with Cirrus or Cirrocumulus.

There are also potentially causal relationships between Cumulus and the other risk scores. Whether the associations between risk scores are causal, and in which direction is unknown; they could also be due to non-causal effects such as confounding and conditioning on colliders [14]. To address the issues of causation, we took a novel approach.

Twin and family studies have shown that the density-based risk scores are correlated between relatives; i.e. they are familial [15]. This means that there could be genetic or non-genetic factors shared by relatives such as twin pairs or sisters that determine these risk scores. Irrespective of the source of such familial determinants, their existence means that we can apply a causal inference method based on data from related pairs called Inference about Causation from Examining FAmiliaL CONfounding (ICE FALCON) [16].

In this study, we applied ICE FALCON to try to understand if the causal relationships between the texture-based mammogram risk score (Cirrus) and three non-overlapping density-related mammogram risk scores (created from Cumulus, Altocumulus, and Cirrocumulus), in order to provide evidence for which risk scores are more relevant to breast cancer aetiology.

## Materials and methods

### Study sample

We used data from the Australian Mammographic Density Twins and Sisters Study [17], which included female twin pairs and their sisters aged 40–70 years and without a prior diagnosis of breast cancer at the time of mammography. Information of the participants was collected by questionnaires, and permissions to access mammograms were obtained [18]. The current study involved 371 monozygotic twin pairs with complete epidemiological information and the mammographic measurements required for analysis. No individual was identified as being at a high risk for breast cancer when taking mammography nor after assessment of their mammograms.

### Questionnaire

Demographic information, anthropometric measurements, menstrual and reproductive history, lifestyle factors, and personal and family history of breast cancer were collected via telephone-administered questionnaires between 2004 and 2008. Zygosity was determined from genome-wide association data [19]. As there were time differences between age at questionnaire survey and age at mammography (on average 1.68 years, with 177 participants having a more than 3-year difference), menopausal status and BMI were updated to those at age at mammography as follows. For menopausal status, if a participant was postmenopausal at questionnaire survey, and her age at menopause was older than age at mammography, her status was changed to premenopausal. BMI at mammography was predicted from BMI at questionnaire survey using the method of Haby et al. [20]. BMI at questionnaire survey was treated as the dependent variable in a regression model which included birth cohort effects and 5-year group coefficients; the intercept of the regression was then used as the BMI at age of mammography.

### Mammogram-based measures

Mammograms were retrieved from BreastScreen Australia services (80%), clinics (5%), and from participants themselves (15%) and digitised using the Lumysis 85 scanner at the Australian Mammographic Density Research Facility. For each woman, only the craniocaudal-view mammogram from the right breast taken closest to the survey was used in this study.

The dense areas were measured using a computer-assisted semi-automated thresholding technique and the CUMULUS software based on a sliding scale ranging from 0 to 4095 pixels. Four observers were trained to measure mammographic density independently, as previously described [4].

A conventional pixel threshold was first used to identify dense areas with grey levels appearing at least mammographically light (the areas of which we call Cumulus). Similarly, the pixel brightness threshold was then increased to identify the denser areas (the areas of which we call Altocumulus). The pixel threshold was then further increased to identify the densest areas (the areas of which we call Cirrocumulus). The reproductivity was assessed by conducting the measurements in sets of 100 mammograms, and 10% of samples in each set were repeated. The intraclass correlation coefficients were 0.98, 0.99, and 0.93 for Cumulus, Altocumulus, and Cirrocumulus, respectively. A total of 200 images were measured for Cumulus, Altocumulus, and Cirrocumulus with the correlations between readers being 0.95, 0.89, and 0.85, respectively. Details of these three density measurements can be found elsewhere [3, 4, 17].

We created two new non-overlapping measures: light areas, which subtracted Altocumulus from Cumulus, and bright areas, which subtracted Cirrocumulus from Altocumulus. Along with a measure of the brightest areas (Cirrocumulus), their relationships, in terms of relative brightness, are shown in Fig. 1.

Cirrus is an agnostic algorithm developed using deep learning techniques applied to 20 textural features extracted from 46,158 analogue, craniocaudal-view, mammograms [8]. The algorithm was applied to the mammograms of the study sample to produce the Cirrus measures.

In this study, we conducted analyses of Cirrus and the three spatially independent density measures including light, bright, and brightest areas (Cirrocumulus). Table 1 shows the summary characteristics of unadjusted measures.

**Table 1:** Characteristics of mammographic measures and covariates of the monozygotic twins

Continuous variables	Mean	Median	25% percentile	75% percentile
Cumulus (cm^2^)	33.15	29.14	18.03	42.68
Altocumulus (cm^2^)	12.37	11.18	6.79	16.10
Brightest areas (Cirrocumulus) (cm^2^)	2.32	1.62	0.75	3.12
Light areas (cm^2^)	20.79	17.40	10.54	26.79
Bright areas (cm^2^)	10.04	8.91	5.81	13.31
Cirrus	2910.32	2910.40	2910.02	2910.75
BMI at mammograms (kg/m^2^)	25.42	24.66	22.21	27.71
Age at mammograms (years)	53.88	53.14	47.45	59.26
Difference between age at mammography and age at survey (years)	1.68	0.23	− 0.43	2.47

Categorical variables	N (%)
*Menopausal status*	
Premenopausal	282 (38%)
Postmenopausal	460 (62%)
*Pregnant*	
Never	85 (11%)
Ever	657 (89%)
*Benign breast disease*	
Never	515 (69%)
Ever	227 (31%)
*Breastfeeding*	
Never	163 (22%)
Ever	579 (78%)
*Number of live births*	
0	102 (14%)
1	57 (8%)
2	260 (35%)
3	206 (28%)
4	88 (12%)
≥5	29 (4%)
*Number of relatives diagnosed with breast cancer*	
0	494 (67%)
1	193 (26%)
2	52 (7%)
3	3 (0%)
*Age at menarche (years)*	
< 12	105 (14%)
≥12 and < 15	508 (69%)
≥15	129 (17%)

### Statistical methods

All mammographic measures were first transformed using a Box–Cox power transformation [21] to have an approximately normal distribution. As a result, (Cirrus-2907)^2^, ${\mathrm{brightest areas }(\mathrm{Cirrocumulus})}^\frac{1}{5},$ and the cube root of light areas and of bright areas were used in the analyses.

Given that age at mammography is negatively associated with the mammographic density measures being studied as putative risk factors for breast cancer, and that breast cancer risk increases with age, all the measures were adjusted for age at mammography. This adjustment explained 8–11% of the variances in the studied measures, except for the light areas, for which the proportion of the variance explained was 2%. The variance explained by other breast cancer risk factors combined, including age at menarche, menopausal status, BMI, ever being pregnant, number of live births, benign breast disease history, and breast cancer family history, was between 4 and 7% (Additional file 1: Table S1).

The age-adjusted residuals were all standardised to have mean = 0 and standard deviation (SD) = 1. These standardised residuals are the mammogram risk scores used in the subsequent analyses. Correlations between these risk scores, within twin pair and within a person, respectively, were estimated using Pearson’s correlation coefficient.

The correlations between the risk scores were decomposed into different sources, including confounding and causal effects originated from various pathways using the Inference about Causation from Examination of FAmilial CONfounding (ICE FALCON) method [16]. ICE FALCON uses data for pairs of relatives and uses the relative’s exposure acts as a proxy instrumental variable for a person’s exposure. This method is analogous to Mendelian randomisation but does not use genetic variants as a presumed instrumental variable and does on rely on strong assumptions. ICE FALCON can make inference about causation even when the exposure and outcome are associated due to familial confounding (i.e. confounders, both known and unknown, that are shared by the exposure and the outcome and by the relatives). The ICE FALCON method has been applied in multiple fields to assess evidence for causality [16, 22–28].

Briefly, one risk score was assigned as the outcome *Y* variable and another as the predictor variable *X*, and the *Y* value of a twin was regressed against the *X* variable of herself and/or of her co-twin (Additional file 1: Figure S1). To assess the evidence for reverse causation, the assigning of *X* and *Y* was reversed, i.e. the aforementioned predictor and outcome swapped their positions in the refitted regression models*.* This was done for every pair of risk scores.

Given the *Y* variables are correlated within twin pairs, regression was conducted using generalised estimating equations. This effect conditioned the *Y* value of a twin on the *Y* value of her co-twin. Our model assumed that the risk score of a twin cannot have a causal effect on the same risk score of her co-twin but allowed for causation between the risk scores within a twin.

Three models were fitted to the twin pair data. First, a twin’s outcome variable was regressed on her own predictor variable to estimate the regression coefficient *β*_self_ (Model 1). Second, the twin’s outcome variable was regressed on her co-twin’s predictor variable to estimate the regression coefficient *β*_co-twin_ (Model 2). Third, the twin’s outcome variable was regressed on both her own and her co-twin’s predictor variables to estimate the conditional regression coefficients *β*^′^_self_ and *β*^′^_co-twin_, respectively (Model 3). The use of the prime on the conditional regression coefficient estimates indicates that the Model 3 regression coefficients can be interpreted as the change in outcome for change in a given predictor while keeping the other predictor constant, which is not the same interpretation for the corresponding unconditional regression coefficients of Models 1 and 2.

If the predictor has a causal effect on the outcome, *β*_co-twin_ would be different from zero, *β*^′^_co-twin_ would be closer to zero than *β*_co-twin_, and *β*^′^_self_ would not be different from *β*_self_. If there is familial confounding between the predictor and the outcome, *β*^′^_self_ and *β*^′^_co-twin_ would both be away from their corresponding coefficients *β*_self_ and *β*_co-twin_ to a similar extent. If there is a combination of familial confounding and causal effects, the results would be the combinations of the two scenarios. According to Wright’s path tracing rules [29], the proportion of an association which could be attributed to causality is as follows:

$$\Pr = ((({\text{Change}}\;{\text{ in}}\; \beta_{{{\text{co}} - {\text{twin}}}} - ({\text{Change }}\;{\text{in}}\; \beta_{{{\text{self}}}} /\beta_{{{\text{self}}}} ) \times \beta_{{{\text{co}} - {\text{twin}}}} )/\rho )/\beta_{{{\text{self}}}} ) \times 100\%$$

where ${\text{Change}}\; {\text{in}}\; \beta_{{{\text{co}} - {\text{twin}}}}$ = $\beta_{{{\text{co}} - {\text{twin}}}} - \beta_{{{\text{co}} - {\text{twin}}}}^{\prime }$, ${\text{Change}}\;{\text{ in}}\; \beta_{{{\text{self}}}}$ = $\beta_{{{\text{self}}}} - \beta_{{{\text{self}}}}^{\prime }$, and $\rho$ = the within-twin correlation of the predictor. Note that the parameter estimates were extracted only from the models which suggest that the predictor causes the outcome, not those that suggest the outcome causes the predictor; see [16]. The causal effect = ${\beta }_{\mathrm{self}}\times \mathrm{Pr}$, so the proportion of an association that could be attributed to familial confounding is 1 − Pr.

To investigate causal pathways between two risk scores that are not through other risk scores, we used the standardised residuals of the predictor and the outcome after adjusting for the third risk score in addition to age at mammography. The results from the analyses were used to produce a summary causal diagram. Causal relationship analyses were also conducted by the level of breast density to check whether the causal relationships differ by density levels. The sample was divided into two subgroups according to the median of 30.5% for Cumulus per cent mammographic density, with each group including 140 complete twin pairs. ICE FALCON analyses were conducted within each subgroup; see Supplemental material for more details. All the analyses were conducted using the R package [30]. *P* < 0.05 was considered to be nominally statistically significant.

## Results

### Correlations between the mammogram risk scores

Table 2 shows that the mammograph risk scores were substantially correlated with each other and within the twin pairs. The within-twin-pair correlations in the risk scores ranged from 0.22 to 0.59. The within-twin cross-trait correlations ranged from 0.28 to 0.81, and the cross-twin cross-trait correlations ranged from 0.28 to 0.61 (all *P* < 0.05).

**Table 2:** The within-twin within-trait correlations, the within-twin cross-trait correlation, and the cross-twin cross-trait correlations between the mammogram risk scores (95% confidence intervals in parentheses)

*X*	*Y*	Within-twin within-trait correlation	Within-twin cross-trait correlation	Cross-twin cross-trait correlation
Light areas	Light areas	0.59 (0.54,0.64) *	–	–
Bright areas	Bright areas	0.53 (0.47,0.58) *	–	–
Brightest areas (Cirrocumulus)	Brightest areas (Cirrocumulus)	0.34 (0.27,0.40) *	–	–
Cirrus	Cirrus	0.48 (0.42,0.53) *	–	–
Light areas ^a^	Light areas ^a^	0.55 (0.50,0.60)	–	–
Bright areas ^a^	Bright areas ^a^	0.41 (0.35,0.47) *	–	–
Brightest areas (Cirrocumulus) ^a^	Brightest areas (Cirrocumulus) ^a^	0.22 (0.15,0.29) *	–	–
Light areas	Bright areas	–	0.81 (0.79,0.84)	0.52 (0.47,0.57)
Light areas	Brightest areas (Cirrocumulus)	–	0.49 (0.44,0.55)	0.35 (0.29,0.41)
Light areas	Cirrus	–	0.38 (0.32,0.44)	0.28 (0.21,0.35)
Bright areas	Brightest areas (Cirrocumulus)	–	0.69 (0.65,0.72)	0.40 (0.34,0.46)
Bright areas	Cirrus	–	0.45 (0.39,0.51)	0.33 (0.26,0.39)
Brightest areas (Cirrocumulus)	Cirrus	–	0.43(0.37,0.49)	0.28 (0.22,0.35)
Bright areas^a^	Brightest areas (Cirrocumulus)^a^	–	0.28 (0.21,0.34)	0.60 (0.57,0.66)
Light areas^a^	Brightest areas (Cirrocumulus)^a^	–	0.27 (0.21, 0.34)	0.39 (0.33, 0.45)

*X* and *Y* represent two traits; *indicates the correlation of the trait within twin pairs; the within-twin within-trait correlations, i.e. *X*_self_ and *X*_co-twin_, or *Y*_self_ and *Y*_co-twin_; the within-twin cross-trait correlations, i.e. *X*_self_ and *Y*_self_, or *X*_co-twin_ and *Y*_co-twin_; and the cross-twin cross-trait correlations, i.e. *X*_self_ and *Y*_co-twin_, or *X*_co-twin_ and *Y*_self_

^a^adjusted for age at mammography and Cirrus

### Causal inference for pairs of mammogram risk scores

Table 3 and Figure S2 (Additional file 1) show the ICE FALCON results and inference about proportions of familial confounding and causation; similar analyses using the Cumulus and Altocumulus measures can be found in Additional file 1: Table S2.

**Table 3:** The relationships between Cirrus and mammographic density measures analysed by using the ICE FALCON method

Assignment of *X*–*Y*		Model 1		Model 2		Model 3		Change*		Conclusion from ICE FALCON	
		Coef. (se)	*P*	Coef. (se)	*P*	Coef. (se)	*P*	Coef. (se)	*P*	Familial confounding	Causal effect
Light areas–bright areas	Self	0.802 (0.030)	2 × 10^–161^			0.770 (0.033)	5 × 10^–117^	0.032 (0.013)	2 × 10^–2^	Yes (11%)	Light areas cause bright areas (89%)
	Co-twin			0.513 (0.041)	4 × 10^–35^	0.070 (0.027)	10^–2^	0.443 (0.037)	8 × 10^–34^		
Bright areas–light areas	Self	0.770 (0.025)	4 × 10^–210^			0.727 (0.026)	10 × 10^–174^	0.043 (0.011)	5 × 10^–5^	Yes (42%)	Bright areas cause light areas (58%)
	Co-twin			0.404 (0.045)	10^–19^	0.144 (0.025)	8 × 10^–9^	0.259 (0.035)	10^–13^		
Light areas–Cirrus	Self	0.352 (0.041)	6 × 10^–18^			0.328 (0.041)	3 × 10^–15^	0.024 (0.011)	2 × 10^–2^	Yes (63%)	Light areas cause Cirrus (37%)
	Co-twin			0.176 (0.038)	4 × 10^–06^	0.087 (0.037)	0.02	0.089 (0.020)	10^–5^		
Cirrus–light areas	Self	0.285 (0.035)	2 × 10^–16^			0.303 (0.034)	6 × 10^–19^	− 0.018 (0.008)	2 × 10^–2^		
	Co-twin			0.091 (0.033)	6 × 10^–03^	0.135 (0.030)	9 × 10^–6^	− 0.045 (0.015)	3 × 10^–3^		
Bright areas–Cirrus	Self	0.398 (0.038)	6 × 10^–26^			0.367 (0.039)	2 × 10^–21^	0.031 (0.010)	2 × 10^–3^	Yes (72%)	Bright areas cause Cirrus (28%)
	Co-twin			0.215 (0.038)	10^–08^	0.139 (0.036)	10^–4^	0.076 (0.021)	2 × 10^–4^		
Cirrus–bright areas	Self	0.361 (0.034)	5 × 10^–26^			0.355 (0.034)	10^–25^	0.006 (0.008)	0.5		
	Co-twin			0.165 (0.036)	6 × 10^–06^	0.163 (0.033)	7 × 10^–7^	0.002 (0.017)	0.9		
Brightest areas (Cirrocumulus)–Cirrus	Self	0.351 (0.040)	2 × 10^–18^			0.356 (0.039)	2 × 10^–20^	− 0.005 (0.008)	0.5	Yes (66%)	Cirrus causes brightest areas (Cirrocumulus) (34%)
	Co-twin			0.138 (0.035)	9 × 10^–05^	0.166 (0.032)	3 × 10^–7^	− 0.028 (0.016)	8 × 10^–2^		
Cirrus–brightest areas (Cirrocumulus)	Self	0.389 (0.039)	10^–23^			0.360 (0.040)	9 × 10^–20^	0.028 (0.010)	4 × 10^–3^		
	Co-twin			0.191 (0.038)	5 × 10^–07^	0.114 (0.036)	2 × 10^–03^	0.077 (0.018)	3 × 10^–5^		
Light areas–brightest areas (Cirrocumulus)	Self	0.470 (0.036)	5 × 10^–38^			0.424 (0.041)	3 × 10^–25^	0.046 (0.017)	8 × 10^–3^	Yes (45%)	Light areas cause brightest areas (Cirrocumulus) (55%)
	Co-twin			0.284 (0.040)	2 × 10^–12^	0.103 (0.037)	5 × 10^–3^	0.181 (0.025)	9 × 10^–13^		
Brightest areas (Cirrocumulus)–light areas	Self	0.347(0.035)	7 × 10^–23^			0.395 (0.033)	4 × 10^–33^	− 0.048 (0.012)	6 × 10^–5^		
	Co-twin			0.113 (0.034)	8 × 10^–4^	0.221 (0.030)	10^–13^	− 0.108 (0.018)	3 × 10^–9^		
Bright areas–brightest areas (Cirrocumulus)	Self	0.680 (0.025)	10^–159^			0.653 (0.029)	3 × 10^–109^	0.027 (0.014)	6 × 10^–2^	Yes (15%)	Bright areas cause brightest areas (Cirrocumulus) (85%)
	Co-twin			0.378 (0.039)	2 × 10^–22^	0.058 (0.031)	7 × 10^–2^	0.320 (0.031)	5 × 10^–25^		
Brightest areas (Cirrocumulus)–bright areas	Self	0.619 (0.032)	2 × 10^–84^			0.606 (0.029)	4 × 10^–94^	0.012 (0.010)	0.2		
	Co-twin			0.136 (0.038)	4 × 10^–4^	0.202 (0.027)	8 × 10^–14^	− 0.065 (0.027)	2 × 10^–2^		
Light areas^a^–brightest areas (Cirrocumulus)^a^	Self	0.386 (0.038)	9 × 10^–25^			0.365 (0.041)	6 × 10^–9^	0.020 (0.016)	0.2	Yes (36%)	Light areas cause brightest areas (Cirrocumulus) not through Cirrus (64%)
	Co-twin			0.198(0.043)	5 × 10^–6^	0.051 (0.040)	0.2	0.147 (0.024)	5 × 10^–10^		
Brightest areas (Cirrocumulus)^a^–light areas^a^	Self	0.287 (0.034)	10^–17^			0.339 (0.034)	2 × 10^–23^	− 0.052 (0.012)	2 × 10^–5^		
	Co-twin			0.046 (0.036)	0.2	0.165 (0.033)	8 × 10^–7^	− 0.119(0.018)	7 × 10^–11^		
Bright areas ^a^–brightest areas (Cirrocumulus)^a^	Self	0.608(0.030)	4 × 10^–90^			0.597 (0.033)	3 × 10^–75^	0.011 (0.011)	0.3	Yes (8%)	Bright areas cause brightest areas (Cirrocumulus) not through Cirrus (92%)
	Co-twin			0.268(0.044)	2 × 10^–9^	0.033 (0.033)	0.3	0.234 (0.032)	2 × 10^–13^		
Brightest areas (Cirrocumulus) ^a^–bright areas^a^	Self	0.562(0.032)	6 × 10^–69^			0.569 (0.031)	3 × 10^–77^	− 0.008 (0.008)	0.3		
	Co-twin			0.058 (0.044)	0.2	0.153 (0.031)	9 × 10^–7^	− 0.096 (0.028)	10^–3^		

se standard error

^a^Cirrus was additionally adjusted

Model 1: *Y*_self_ = *β*_self_*X*_self_ + *ε*_1_, Model 2: *Y*_self_ = *β*_co-twin_*X*_co-twin_ + *ε*_2_, and Model 3: *Y*_self_ = *β*_self_*X*_self _+ *β*_co-twin_*X*_co-twin_ + *ε*_3_

*Change refers to the change in coefficients from Model 1 to Model 3 for a woman herself, the change in coefficients from Model 2 to Model 3 for the woman’s co-twin

### The bright areas and light areas

With the light areas as the predictor and the bright areas as the outcome, there was a decrease of 4% (*P* = 0.02) from *β*_self_ = 0.802 (*P* = 10^–161^) in Model 1 to *β*^′^_self_ = 0.770 (*P* = 10^–117^) in Model 3 and a decrease of 86% (*P* = 10^–34^) from *β*_co-twin =_ 0.513 (*P* = 10^–34^) in Model 2 to *β*^′^_co-twin_ = 0.070 (*P* = 0.01) in Model 3. These results were consistent with the light areas having a causal effect on the bright areas that accounted for 89% of their association, with marginal evidence for familial confounding.

With the bright areas as the predictor and the light areas as the outcome, there was a decrease of 6% (*P* = 10^–5^) from *β*_self_ = 0.770 (*P* = 10^–210^) in Model 1 to *β*^′^_self_ = 0.727 (*P* = 10^–174^) in Model 3 and a decrease of 64% (*P* = 10^–33^) from *β*_co-twin_ = 0.404 (*P* = 10^–19^) in Model 2 to *β*^′^_co-twin_ = 0.144 (*P* = 10^–9^) in Model 3. These results were consistent with the bright areas having a causal effect on the light areas that accounted for 58% of their association and familial confounding that accounted for 42%.

Therefore, the ICE FALCON results suggest the existence of familial confounding and bidirectional causality between the light areas and bright areas. To avoid confusion arising from the potential bidirectional causation, we conducted analyses separately for the light areas and bright areas.

### Cirrus and the light areas

With Cirrus as the outcome and the light areas as the predictor, there was a decrease of 7% (*P* = 0.03) from *β*_self_ = 0.352 (*P* = 10^–17^) in Model 1 to *β*^′^_self_ = 0.328 (*P* = 10^–16^) after adjusting for co-twin’s light areas in Model 3. There was a decrease of 51% (*P* = 10^–7^) from *β*_co-twin_ = 0.176 (*P* = 10^–5^) in Model 2 to *β*^′^_co-twin_ = 0.087 (*P* = 0.02) after adjusting for the twin’s light areas in Model 3. These results are consistent with there being a combination of familial confounding that accounted for 37% of the association between the two risk scores as well as the light areas having a causal effect on Cirrus that accounted for 63% of their association.

We then reversed the predictor and outcome roles by assigning the light areas to be the outcome and Cirrus as the predictor. There was an increase from *β*_self_ = 0.285 (*P* = 10^–15^) in Model 1 to *β*^′^_self_ = 0.303 (*P* = 10^–20^) in Model 3 (*P* = 0.02). There was also an increase in the co-twin’s coefficient from *β*_co-twin_ = 0.091 (*P* = 0.006) in Model 2 to *β*^′^_co-twin_ = 0.135 (*P* = 10^–5^) in Model 3 (*P* = 0.003). These results were consistent with the findings above that the association was due to a combination of familial confounding and the light areas having a causal effect on Cirrus.

### Cirrus and the bright areas

With Cirrus as the outcome and the bright areas as the predictor, there was a decrease of 8% (*P* = 0.002) from *β*_self_ = 0.398 (*P* = 10^–27^) in Model 1 to *β*^′^_self_ = 0.367 (*P* = 10^–21^) in Model 3. There was a decrease of 35% (*P* = 10^–4^) from *β*_co-twin_ = 0.215 (*P* = 10^–8^) in Model 2 to *β*^′^_co-twin_ = 0.139 (*P* = 10^–4^) in Model 3. These results were consistent with there being a combination of familial confounding that accounted for 72% of the association between the two risk scores, and the bright areas having a causal effect on Cirrus that accounted for 28% of their association.

When we reversed the predictor and outcome roles by assigning the bright areas to be the outcome and Cirrus as the predictor, *β*_self_ was not significantly different from *β*^′^_self_ after adjusting for co-twin’s Cirrus (*P* = 0.5), and a similar statement applies to the lack of a difference between *β*_co-twin_ and *β*^′^_co-twin_ (*P* = 0.9). These results were not consistent with Cirrus having a causal effect on the bright areas.

### Cirrus and the brightest areas (Cirrocumulus)

With brightest areas (Cirrocumulus) as the outcome and Cirrus as the predictor, there was a decrease of 7% (*P* = 10^–3^) from *β*_self_ = 0.389 (*P* = 10^–23^) in Model 1 to *β*^′^_self_ = 0.360 (*P* = 10^–19^) in Model 3 (*P* = 0.004), while there was a decrease of 40% (*P* = 10^–5^) from *β*_co-twin_ = 0.191 (*P* = 10^–6^) in Model 2 to *β*^′^_co-twin_ = 0.114 (*P* = 0.002) in Model 3 (*P* = 10^–5^). These results were consistent with Cirrus having a causal effect on the brightest areas (Cirrocumulus), which accounted for 34% of their association, as well as there being familial confounding which accounted for 64%.

When we reversed the predictor and outcome roles by assigning brightest areas (Cirrocumulus) to be the predictor and Cirrus as the outcome, there was no difference between *β*_self_ and *β*^′^_self_ (*P* = 0.5), while there was an increase from *β*_co-twin =_ 0.138 (*P* = 10^–5^) in Model 2 to *β*^′^_co-twin_ = 0.166 (*P* = 10^–7^) in Model 3 that was not nominally significant (*P* = 0.08). These results were not consistent with the brightest areas (Cirrocumulus) having a causal effect on Cirrus.

### The light areas and brightest areas (Cirrocumulus)

With the light areas as the predictor and the brightest areas (Cirrocumulus) as the outcome, there was a decrease of 10% (*P* = 0.008) from *β*_self_ = 0.470 (*P* = 10^–38^) in Model 1 to *β*^′^_self_ = 0.424 (*P* = 10^–25^) in Model 3 and a decrease of 64% (*P* = 10^–13^) from *β*_co-twin_ = 0.284 (*P* = 10^–12^) in Model 2 to *β*^′^_co-twin_ = 0.103 (*P* = 0.005) in Model 3. These results were consistent with the light areas having a causal effect on the brightest areas (Cirrocumulus) that accounted for 55% of their association and familial confounding which accounted for 45%.

With the brightest areas (Cirrocumulus) as the predictor and the light areas as the outcome, there was an increase from *β*_self_ = 0.347 (*P* = 10^–23^) in Model 1 to 0.395 (*P* = 10^–33^) in Model 3 (*P* = 10^–5^) and an increase from *β*_co-twin_ = 0.113 (*P* = 10^–4^) in Model 2 to *β*^′^_co-twin_ = 0.221 (*P* = 10^–13^) in Model 3 (*P* = 10^–9^). These results were not consistent with the brightest areas (Cirrocumulus) having a causal effect on the light areas.

### The bright areas and brightest areas (Cirrocumulus)

With the bright areas as the predictor and the brightest areas (Cirrocumulus) as the outcome, there was a marginally significant decrease of 4% (*P* = 0.06) from *β*_self_ = 0.680 (*P* = 10^–159^) in Model 1 to *β*^′^_self_ = 0.653 (*P* = 10^–109^) in Model 3 (*P* = 0.06), while there was a decrease of 85% (*P* = 10^–25^) from *β*_co-twin_ = 0.378 (*P* = 10^–22^) in Model 2 to *β*^′^_co-twin_ = 0.058 (*P* = 0.07) in Model 3 of 85% (*P* = 10^–25^). These results were consistent with the bright areas having a causal effect on the brightest areas (Cirrocumulus) that accounted for 85% of their association and familial confounding which accounted for 15%.

With the brightest areas (Cirrocumulus) as the predictor and the bright areas as the outcome, there was no difference between *β*_self_ and *β*^′^_self_ (*P* = 0.21), while there was an increase in *β*_co-twin_ = 0.136 (*P* = 10^–4^) in Model 2 to *β*^′^_co-twin_ = 0.202 (*P* = 10^–14^) in Model 3 (*P* = 0.02). These results were not consistent with the brightest areas (Cirrocumulus) having a causal effect on the bright areas.

The above results were consistent with the existence of causal pathways from both the light areas and the bright areas to the brightest areas (Cirrocumulus), and to Cirrus, and from Cirrus to the brightest areas (Cirrocumulus).

### Causal inference for the light areas and brightest areas (Cirrocumulus) not through Cirrus

With the light areas adjusted for Cirrus as the predictor and the brightest areas (Cirrocumulus) adjusted for Cirrus as the outcome, there was no difference between *β*_self_ in Model 1 and *β*^′^_self_ in Model 3 (*P* = 0.2), while there was a decrease from *β*_co-twin_ = 0.198 (*P* = 10^–6^) in Model 2 to *β*^′^_co-twin_ = 0.051 (*P* = 0.2) in Model 3 by 74% (*P* = 10^–10^). These results were consistent with a causal effect from the light areas to the brightest areas (Cirrocumulus) that not through Cirrus, which accounted for 64% of their association and with familial confounding that accounted for 36%.

With brightest areas (Cirrocumulus) adjusted for Cirrus as the predictor and light areas adjusted for Cirrus as the outcome, *β*_self_ increased from 0.287 (*P* = 10^–17^) in Model 1 to 0.339 (*P* = 10^–23^) after adjusting for co-twin’s brightest areas (Cirrocumulus) in Model 3 (*P* = 10^–5^), *β*_co-twin_ increased from 0.046 (*P* = 0.2) in Model 2 to *β*^′^_co-twin_ = 0.165 (*P* = 10^–7^) after adjusting for the twin’s brightest areas (Cirrocumulus) in Model 3 (*P* = 10^–11^). These results were consistent with a combination of a causal effect from the light areas to the brightest areas (Cirrocumulus) not through Cirrus and familial confounding.

### Causal inference for the bright areas and brightest areas (Cirrocumulus) not through Cirrus

With the bright areas adjusted for Cirrus as the predictor and the brightest areas (Cirrocumulus) adjusted for Cirrus as the outcome, *β*_self_ decreased, but not significantly (*P* = 0.3), from 0.608 (*P* = 10^–90^) in Model 1 to *β*^′^_self_ = 0.597 (*P* = 10^–75^) after adjusting for co-twin’s bright areas in Model 3, *β*_co-twin_ decreased from 0.268 (*P* = 10^–9^) in Model 2 to the null (*P* = 0.3) after adjusting for co-twin’s bright areas in Model 3 by 87% (*P* = 10^–13^). These results were consistent with the bright areas having a causal effect on the brightest areas (Cirrocumulus) not through Cirrus, which accounted for 92% of their association and the familial confounding accounting for 8% of the association.

With the brightest areas (Cirrocumulus) adjusted for Cirrus as the predictor and the bright areas adjusted for Cirrus as the outcome, *β*_self_ did not change significantly (*P* = 0.3) after adjusting for co-twin’s brightest areas (Cirrocumulus), *β*_co-twin_ increased from 0.08 (*P* = 0.186) in Model 2 to *β*^′^_co-twin_ 0.153 (*P* = 10^–6^) after adjusting for the twin’s brightest areas (Cirrocumulus) in Model 3 (*P* = 0.001). These results are consistent with a combination of a causal effect, from the bright areas to the brightest areas (Cirrocumulus) not through Cirrus, and familial confounding.

From the subgroup analyses according to the level of per cent mammographic density based on Cumulus measure, similar causal evidence was found between subgroups for the causal relationships between most pairs of mammographic risk scores, which supported that the causal relationships were unlikely to depend on breast density level (Additional file 1: Tables S3 and S4). However, the reduced sample size in subgroups might limit the statistical power to detect a difference.

**Fig. 2:**
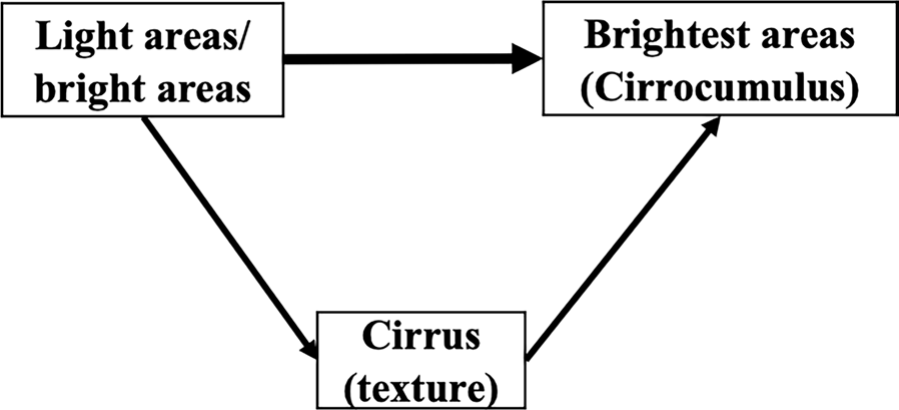
Diagrammatic representation of the inferred causal pathways between the Cirrus risk score and the risk scores based in the spatially independent mammographic density measures: light areas, bright areas, and brightest areas. Note: The thickness of the lines represents the relative strength of the inferred causal effects. For simplicity, the familial confounding between the pairs of risk scores is not shown in the diagram

Figure 2 shows the possible causal pathways between the three mammographic density measures and Cirrus, based on the results presented.

## Discussion

This study has shown that the amount of lighter (less dense) areas a woman has on her mammogram might cause her to also have more of the brightest (highly dense) areas, currently the strongest density measure associated with breast cancer risk [2–4, 7–9]. This causal relationship could be direct, but it could also be through the amount of less dense area having a causal effect on specific textural features that are themselves associated with breast cancer risk.

Our findings as encapsulated by Fig. 2 could also explain the observations from a recent publication of the WECARE study that the associations of contralateral breast cancer risk with the light areas and bright areas were attenuated to the null after adjusting for the brightest areas (Cirrocumulus), while the association with the brightest areas (Cirrocumulus) remained unchanged; the risk gradients for the light, bright, and brightest areas (Cirrocumulus) were 1.24, 1.34, and 1.40 when fitted alone, were 0.98, 1.01, and 1.40 when fitted together, respectively [6]. That is the associations of the light and bright areas with contralateral breast cancer risk could be due to their causal associations with the brightest areas (Cirrocumulus); the brightest areas (Cirrocumulus) are more aetiologically important than the light and bright areas in the causal pathway for contralateral breast cancer.

Note that when the light areas risk score was replaced by Cumulus (i.e. the areas including light, bright, and brightest areas (Cirrocumulus)), and the bright areas risk score was replaced by Altocumulus (i.e. the areas including bright and brightest areas (Cirrocumulus)) in the above models, the results were similar [6]. This is reasonable, because Cumulus is predominated by the light areas as well as bright areas (see Table 1), Altocumulus is predominated by the bright areas, and both the light and bright areas have similar causal relationships with the brightest areas (Cirrocumulus). Similar results were also observed for the associations of Cumulus, the brightest areas (Cirrocumulus), and/or Altocumulus with screen-detected and young-age-at-diagnosis breast cancer, respectively [5, 9, 10]. Conversely, the association of interval breast cancer with the brightest areas (Cirrocumulus) was attenuated to the null when adjusting for Cumulus [10]. Therefore, as what was observed from WECARE study for contralateral breast cancer, the brightest areas (Cirrocumulus) are more aetiologically important than lighter areas for screen-detected and young-age-at-diagnosis breast cancer, while the light areas may have independent causal effects on interval breast cancer, distinct from brightest areas (Cirrocumulus). It is worth noting that the risk associations between interval cancer and the brightest areas (Cirrocumulus) could be due to confounding caused by the light areas.

Subtracting the causal pathways involving Cirrus, the causal effect of bright areas on brightest areas (Cirrocumulus) remained strong, while the causal effect from light areas on brightest areas (Cirrocumulus) was much weaker (i.e. effect size of 0.3 versus 0.6). This is consistent with the observations that the greater the brightness of the dense region the stronger its association with breast cancer risk [6, 10].

For Cirrus, the effect size of its causal associations with density measures is around 0.1, regardless of the direction and the threshold for defining dense areas. Dense area-based measures all have positive associations with Cirrus. Of them, bright areas and light areas are both the causes; while brightest areas (Cirrocumulus) are causally affected by Cirrus, which is less aetiologically important than light areas and bright areas, with both of their total effect sizes larger than 0.2. The weak causal relationships between Cirrus and other mammogram risk scores are reasonable. Apart from Cirrus capturing textural information (i.e. various patterns identified by considering the spatial relationships between intensity levels), which is different from what can be captured using the threshold-based measures (i.e. pixel counts above or below an intensity level), Cirrus also uses the information from the whole breast, rather than the local information measured by threshold-based measures. These results add grounded evidence to previous association-based speculations that texture-based mammographic measures, at least Cirrus, could be an independent and intrinsic risk factor for breast cancer [7, 12].

The findings of this study could inform biological research in trying to understand why conventional density is associated with breast cancer risk. Mammographic density is defined by different levels of pixel brightness thought to represent different types of breast tissue based on their differential X-ray attenuation. Fat tissue appears to be radiologically dark, while fibroglandular tissue appears to be light or bright. As mammograms are two-dimensional representations of tissue composition, each pixel in the image reflects a combination of fibroglandular and fat tissue. Malignant breast tissue, however, is developed from fibroglandular tissue and appears radiologically brighter than normal fibroglandular tissue. Therefore, the brightest areas (Cirrocumulus) contain more pixels and are closer to the radiological appearance of malignant breast tissue than the light areas and bright areas. This might explain in part its typically stronger association with screen-detected, young-age-at-diagnosis, and contralateral breast cancer risk, than other mammographic density measures, as observed in the previous studies [6, 10]. It could also explain why the risk associations of conventional density measures were attenuated once the brightest areas (Cirrocumulus) were included [6, 10].

Additionally, light areas contain a greater amount of less dense tissue (dispersed fibroglandular tissue and possibly fat tissue), compared with bright areas. The difference in tissue composition might explain the observed bidirectional causal relationships between light areas and bright areas, with the causal effect size of 0.7 on bright areas caused by light areas being stronger than the reverse causal effect size of 0.4. Therefore, less dense tissue, including dispersed fibroglandular tissue or fat tissue, could have causal effects on dense fibroglandular tissue.

The study has also demonstrated the value of ICE FALCON in decomposing associations between intercorrelated disease biomarkers into pathways. These causal associations could be identified and quantified even though there was also familial confounding, something that by definition cannot be considered if we used Mendelian randomisation based on assuming genetic variants for these mammogram risk scores were true instrumental variable. The same approach could be applied to other biomarkers and diseases, such as lipids and cardiovascular diseases.

One strength of our study is that when conducting the ICE FALCON analyses, which can infer causation and its direction even if there is a familial confounding between mammogram risk scores, we used monozygotic twin pairs. This maximised the within-pair correlations in the risk scores, and they were close to 0.5. This also maximised the potential existence of non-genetic factors shared by twins and hence the amount of familial confounding.

Considering that there could be bidirectional causal relationships between two traits and the need to make robust inference, we conducted ICE FALCON analyses by switching the roles of predictors and outcome for each pair. Causal inference was only made when the evidence from the two rounds of analyses was consistent or does not contradict each other. We also extensively checked the influence of other common covariates, on the mammographic measures included in this study, that have established or possible associations with breast cancer risk or mammographic density measures according to the previous publications.

One limitation of the study is that the epidemiological data were not collected strictly contemporaneous with the participants’ mammography episodes. But we have previously shown that mammographic density adjusted for age and/or BMI tracks strongly with time, at least over 8 years [31, 32]. Also, the time difference was small (on average 1.68 years, with 177 participants having a more than 3-year difference), and we updated the menopausal status and BMI based on the date information from the questionnaires and mammograms; we consider that the influence on our conclusions due to the time difference should be minimal. Another limitation is that our analyses were based on film mammograms rather than digital mammograms, which have now replaced film mammograms and yield some different image characteristics [33]. We are currently working to try to replicate the study findings using digital mammograms. Given our research on different definitions of density based on brightness found similar results for digitised and digital mammograms [3, 4] in terms of breast cancer risk prediction, we expect the associations and relevant causal estimates between the three dense areas are similar between digitised and digital mammograms. The presence of measurement error in our study poses another limitation, as the assessment of dense areas relies on human measurement. Measurement error in the mammogram risk scores is expected to bias the associations between the risk scores and estimates for familial confounding and causal effects towards the null. However, considering the high reproducibility of the measures in our study (see Methods), the measurement error is negligible; therefore, the influence of measurement error on our results is likely to be minimal.

To the best of our knowledge, we are the first to investigate causal relationships between mammographic measures that predict breast cancer risk. The previous studies on mammographic density have primarily focused on its risk prediction capabilities for breast cancer [34, 35] with an emphasis on conventional mammographic density, and twin and family studies mainly for investigating familial aggregation and heritability of the density measure [15, 18, 19, 36–38]. In contrast, our study takes a novel approach by applying a twin-based design to make causal inference in cross-sectional data. Specifically, we utilise the within- and cross-twin relationships between two variables and apply the analytic method of ICE FALCON. We are also the creators of the mammographic density measures defined by the increasing threshold of pixel brightness.

## Conclusions

In a mammograme, the less dense (light and bright) areas could have a positive causal effect on the densest (brightest) areas which eventually become cancers themselves. There could also be another pathway to cancer evident through textural features not related to density per se, i.e. Cirrus, which also has a causal effect on the brightest areas (Cirrocumulus). Given our previous findings, the brightest areas (Cirrocumulus) are more aetiologically important than lighter areas for screen-detected, young-age-at-diagnosis, and contralateral breast cancer, while light areas have an independent causal effect from the brightest areas for interval breast cancer. In addition to the causal effects from density measures, mammographic measures based on specific textural features, like Cirrus, contain aetiologically independent information for breast cancer risk [6, 10].

### Supplementary Information

**Additional file 1: Figure S1. **The diagram of ICE FALCON methodology. **Figure S2.** The diagram of the relationships between risk scores analysed using ICE FALCON. **Table S1.** Linear mixed-effects models of mammographic measures and covariates. **Table S2.** The relationships between Cirrus and mammographic density measures analysed by using the ICE FALCON method. **Table S3.** The relationships between Cirrus and mammographic density measures for percent mammographic density ≤ 30.5% analysed by using the ICE FALCON method. **Table S4.** The relationships between Cirrus and mammographic density measures for percent mammographic density > 30.5% analysed by using the ICE FALCON method.

## Data Availability

The datasets used and/or analysed during the current study are available from the corresponding author on reasonable request.

## Abbreviations

BMI: Body mass index
ICE FALCON: Inference about causation from examination of familial confounding
SD: Standard deviation
