# Supplementary material for: Causal relationships between breast cancer risk factors based on mammographic features
Source: Breast Cancer Res. 2023 Oct 25;25:127. doi: 10.1186/s13058-023-01733-1 (PMC10598934; doi:10.1186/s13058-023-01733-1)
Supplement: Supplementary file 1 — Additional file 1: Figure S1. The diagram of ICE FALCON methodology. Figure S2. The diagram of the relationships between risk scores analysed using ICE FALCON. Table S1. Linear mixed-effects models of mammographic measures and covariates. Table S2. The relationships between Cirrus and mammographic density measures analysed by using the ICE FALCON method. Table S3. The relationships between Cirrus and mammographic density measures for percent mammographic density ≤ 30.5% analysed by using the ICE FALCON method. Table S4. The relationships between Cirrus and mammographic density measures for percent mammographic density > 30.5% analysed by using the ICE FALCON method. [file 13058_2023_1733_MOESM1_ESM.docx]

**Supplementary material**


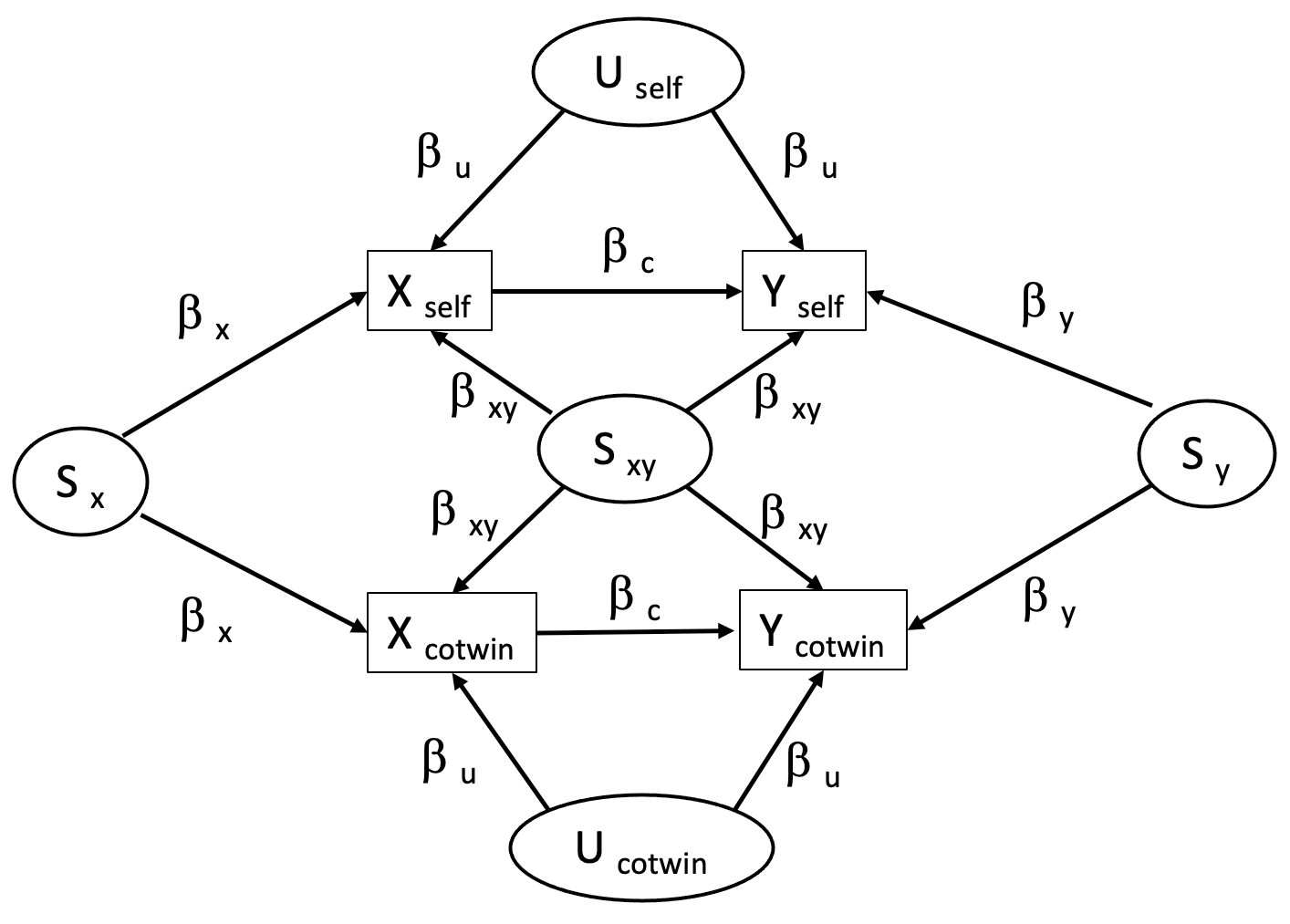


**Figure S1 The diagram of ICE FALCON methodology**

This diagram assumes for a twin pair, the association between two risk scores (X and Y) is due to a causal effect of X on Y, familial confounders shared by the twins and two risk scores (S_XY_), and individual-specific confounders (U_self_ and U_co-twin_). Any observed individual-specific confounder has been controlled in the analysis. S_X_ and S_Y_ represent the determinants of X and Y, respectively, that are shared by the twins. β represents the coefficient along the path.


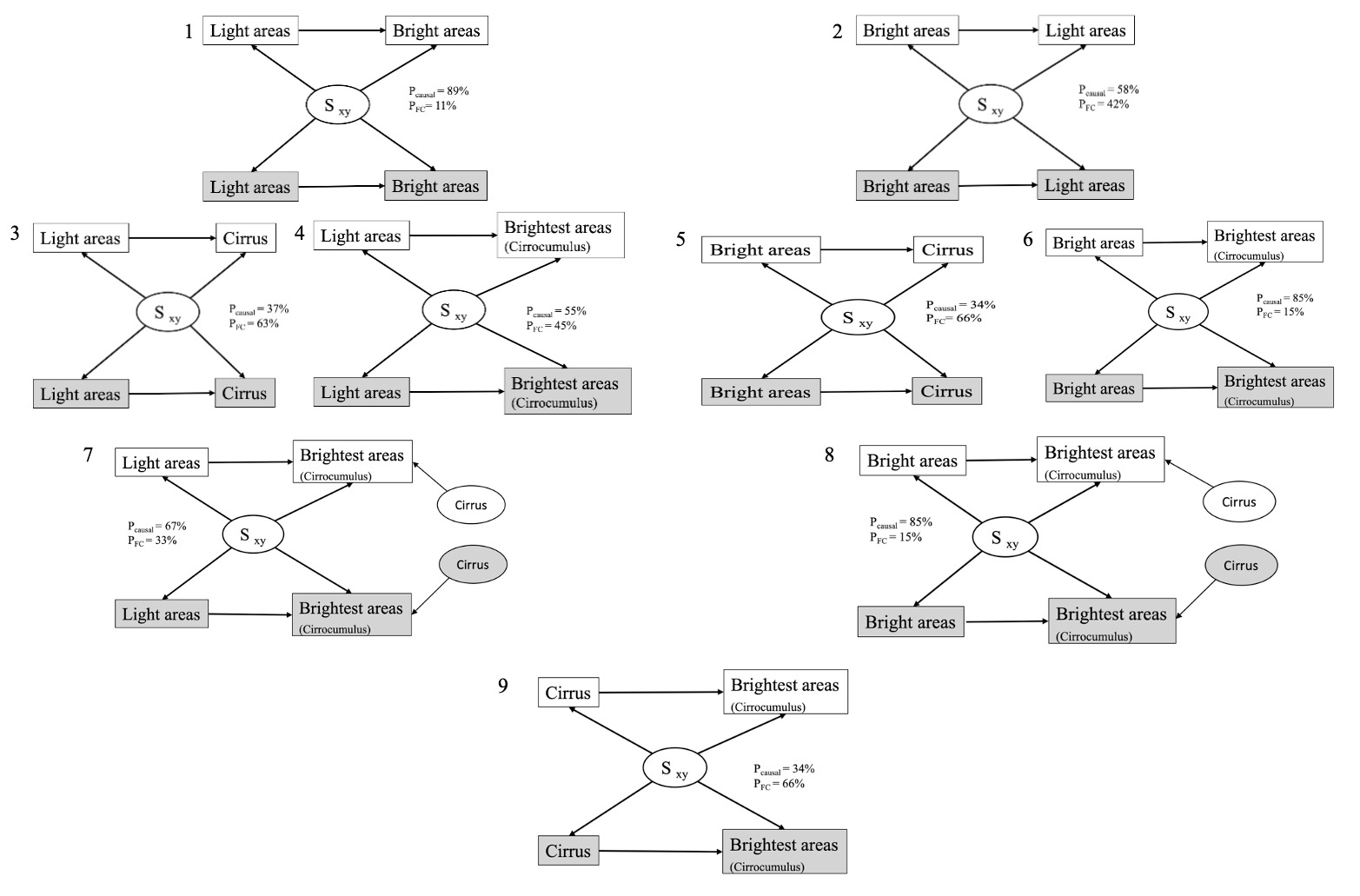


Figure S2 The diagram of the relationships between risk scores analysed using ICE FALCON

Figure S2_1, 3, 4, and 7 illustrate the causal relationships between the light areas and bright areas, light areas and Cirrus, light areas and the brightest areas, and light areas and the bright areas adjusting for Cirrus, respectively. Figure S2_2, 5, 6, and 8 illustrate the causal relationships between bright areas and light areas, bright areas and Cirrus, bright areas and the brightest areas, and bright areas and the brightest areas adjusting for Cirrus, respectively. Figure S2_9 illustrates the causal relationship between Cirrus and the brightest areas.

The influence of covariates included in this paper on the four mammographic measures were checked by conducting two linear mixed-effects models for each mammographic measure (Table S1). In Model 1, the covariates that had a P-value of the association with the dependent variable less than 0.1 when adjusting for other covariates were retained. In Model 2, age at mammograms was excluded from Model 1.

In Model 1, the covariates explained 11-16% of the variation of Cirrus, bright areas, and brightest areas, 6% of light areas. In Model 2, after excluding age at mammograms from all the models, the decreases in R^2^ were 7-10% for Cirrus, bright areas, and brightest areas, was 2% for light areas.

Table S2 shows the result of causal relationships between Cirrus and density measures based on non-overlapping dense areas by using ICE FALCON method. The evidence is consistent with the conclusion that there is a causal effect from Cumulus on Cirrus, accounting for 43% of their association, and the rest 57% is explained by familial confounding; there is a causal effect from Altocumulus on Cirrus, accounting for 30% of their association, and the rest 70% is explained by familial confounding; there is bi-directional causal relationships between the light areas and Altocumulus: for one direction, 97% of the association is due to acausal effect from light areas on Altocumulus, with the rest being due to familial confounding; for the other direction, 58% of the association is due to a causal effect from Altocumulus on the light areas, with 42% being due to familial confounding.

Causal relationship analyses were also conducted by the level of breast density to check whether the causal relationships differ by density levels. The sample was divided into two subgroups according to the median of 30.5% for Cumulus percent mammographic density, with each group including 140 complete twin pairs. ICE FALCON analyses were conducted within each subgroup.

Table S3 and S4 show the subgroup-specific ICE FALCON results. For most of the pairs of mammographic risk scores, the same causal evidence was found between the two subgroups, and the causal evidence was consistent with those found using the whole sample. Note that, the power of this analysis is reduced due to the reduced sampled size in each subgroup.

| **Table S1. Linear mixed-effects models of mammographic measures and covariates** | | | | | |  |
| --- | --- | --- | --- | --- | --- | --- |
|  | **Model 1** |  |  | **Model 2** |  | |
| **Covariates** | **Est(se)** | ***P*** |  | **Est(se)** | ***P*** | |
| **Dependent variable: Cirrus** | | | | | |  |
| Breastfeed ever | 0.57(0.34) | 0.094 |  | 0.75(0.35) | 0.030 | |
| Live birth number | -0.29(0.11) | 0.012 |  | -0.36(0.12) | 0.002 | |
| Breast cancer family history number | 0.39(0.23) | 0.084 |  | 0.44(0.23) | 0.061 | |
| 1/BMI | 35.63(19.71) | 0.071 |  | 52.78(20.14) | 0.009 | |
| Benign breast disease history | 0.73(0.26) | 0.005 |  | 0.60(0.26) | 0.022 | |
| Age at mammograms | -0.12(0.02) | 5×10^-11^ |  |  |  | |
| R^2^ | 0.11 |  |  | 0.04 |  | |
|  |  |  |  |  |  | |
| **Dependent variable: light areas** | | | | | |  |
| Age at menarche | 0.07(0.04) | 0.060 |  | 0.06(0.04) | 0.090 | |
| Live birth number | -0.07(0.02) | 10^-4^ |  | -0.07(0.02) | 4×10^-5^ | |
| Breast cancer family history number | 0.11(0.04) | 0.007 |  | 0.11(0.04) | 0.006 | |
| Benign breast disease history | 0.11(0.04) | 0.004 |  | 0.11(0.04) | 0.008 | |
| Breastfeed ever | 0.10(0.05) | 0.060 |  | 0.11(0.05) | 0.038 | |
| Age at mammograms | -0.01(0.00) | 0.001 |  |  |  | |
| R^2^ | 0.06 |  |  | 0.04 |  | |
|  |  |  |  |  |  | |
| **Dependent variable: bright areas** | | | | | |  |
| Live birth number | -0.02(0.01) | 0.048 |  | -0.03(0.01) | 0.024 | |
| Breast cancer family history number | 0.09(0.03) | 0.002 |  | 0.09(0.03) | 0.001 | |
| 1/BMI | 6.64(2.37) | 0.005 |  | 8.96(2.44) | 3×10^-4^ | |
| Benign breast disease history | 0.15(0.03) | 4×10^-7^ |  | 0.14(0.03) | 10^-5^ | |
| Age at mammograms | -0.02(0.00) | 3×10^-13^ |  |  |  | |
| R^2^ | 0.15 |  |  | 0.07 |  | |
|  |  |  |  |  |  | |
| **Dependent variable: brightest areas** | | | | | |  |
| Live birth number | -0.01(0.01) | 0.012 |  | -0.02(0.01) | 0.004 | |
| Breast cancer family history number | 0.03(0.01) | 0.056 |  | 0.03(0.01) | 0.040 | |
| Benign breast disease history | 0.06(0.02) | 4×10^-4^ |  | 0.05(0.02) | 0.006 | |
| 1/BMI | 4.37(1.19) | 3×10^-4^ |  | 5.61(1.23) | 7×10^-6^ | |
| Age at mammograms | -0.01(0.00) | 10^-15^ |  |  |  | |
| R^2^ | 0.16 |  |  | 0.06 |  | |

se: standard error

**Table S2.** **The relationships between Cirrus and mammographic density measures analysed by using the ICE FALCON method**

|  |  | **Model 1** |  | **Model 2** |  | **Model 3** |  | **Change** |  | **Conclusion from ICE FALCON** | |
| --- | --- | --- | --- | --- | --- | --- | --- | --- | --- | --- | --- |
| **X-Y** |  | **Coef.(se)** | ***P*** | **Coef.(se)** | ***P*** | **Coef.(se)** | ***P*** | **Coef.(se)** | ***P*** | **Familial confounding** | **Causal effect** |
| Cumulus-Cirrus | Self | 0.406(0.040) | 1.E-23 |  |  | 0.379(0.042) | 8.E-20 | 0.027(0.012) | 3.E-02 | Yes (57%) | Cumulus causes Cirrus (43%) |
|  | Co-twin |  |  | 0.206(0.039) | 1.E-07 | 0.084(0.038) | 3.E-02 | 0.121(0.023) | 2.E-07 |  |  |
| Cirrus-Cumulus | Self | 0.319(0.033) | 3.E-22 |  |  | 0.342(0.032) | 3.E-26 | -0.022(0.009) | 1.E-02 |  |  |
|  | Co-twin |  |  | 0.089(0.034) | 8.E-03 | 0.149(0.030) | 8.E-07 | -0.060(0.017) | 5.E-04 |  |  |
|  |  |  |  |  |  |  |  |  |  |  |  |
| Altocumulus-  Cirrus | Self | 0.423(0.038) | 3.E-29 |  |  | 0.393(0.039) | 3.E-24 | 0.030(0.010) | 2.E-03 | Yes (70%) | Altocumulus causes Cirrus (30%) |
|  | Co-twin |  |  | 0.211(0.038) | 2.E-08 | 0.130(0.036) | 4.E-04 | 0.082(0.021) | 9.E-05 |  |  |
| Cirrus-  Altocumulus | Self | 0.380(0.035) | 1.E-27 |  |  | 0.376(0.035) | 3.E-27 | 0.004(0.008) | 0.6 |  |  |
|  | Co-twin |  |  | 0.149(0.037) | 5.E-05 | 0.154(0.033) | 3.E-06 | -0.005(0.018) | 0.8 |  |  |
|  |  |  |  |  |  |  |  |  |  |  |  |
| Light areas-  Altocumulus | Self | 0.759(0.031) | 6.E-132 |  |  | 0.723(0.034) | 4.E-98 | 0.036(0.013) | 6.E-03 | Yes (3%) | Light areas cause Altocumulus (97%) |
|  | Co-twin |  |  | 0.442(0.043) | 1.E-24 | 0.084(0.028) | 3.E-03 | 0.358(0.036) | 8.E-24 |  |  |
| Altocumulus-  light areas | Self | 0.727(0.026) | 2.E-167 |  |  | 0.687(0.027) | 9.E-139 | 0.040(0.011) | 2.E-04 | Yes (42%) | Altocumulus causes light areas (58%) |
|  | Co-twin |  |  | 0.329(0.044) | 1.E-13 | 0.155(0.026) | 2.E-09 | 0.174(0.033) | 1.E-07 |  |  |

se standard error

Model 1: Y_self_ = β_self_X_self_ + ε_1_, Model 2: Y_self_ = β_cotwin_X_cotwin_ + ε_2_, Model 3: Y_self_ = β_self_X_self_ + β_cotwin_X_cotwin_ + ε_3_

*change refers to the change in coefficients from Model 1 to Model 3 for woman herself, the change in coefficients from Model 2 to Model 3 for woman’s cotwin

**Table S3 The relationships between Cirrus and mammographic density measures for percent mammographic density ≤ 30.5% analysed by using the ICE FALCON method**

| **Assignment**  **of X-Y** |  | **Model 1** |  | **Model 2** |  | **Model 3** |  | **Change*** |  | **Causal relationship conclusion** |
| --- | --- | --- | --- | --- | --- | --- | --- | --- | --- | --- |
|  |  | **Coef. (se)** | ***P*** | **Coef. (se)** | ***P*** | **Coef. (se)** | ***P*** | **Coef. (se)** | ***P*** |  |
| Light areas-bright areas | Self | 0.682(0.069) | 7.E-23 |  |  | 0.651(0.075) | 3.E-18 | 0.031(0.023) | 0.2 | Light areas cause bright areas |
|  | Co-twin |  |  | 0.489(0.062) | 5.E-15 | 0.077(0.055) | 0.2 | 0.412(0.054) | 2.E-14 |  |
| Bright areas-light areas | Self | 0.631(0.047) | 6.E-42 |  |  | 0.610(0.042) | 6.E-47 | 0.021(0.017) | 0.2 | Bright areas cause light areas |
|  | Co-twin |  |  | 0.361(0.070) | 3.E-07 | 0.168(0.043) | 8.E-05 | 0.193(0.054) | 3.E-04 |  |
|  |  |  |  |  |  |  |  |  |  |  |
| Light areas-Cirrus | Self | 0.300(0.079) | 1.E-04 |  |  | 0.282(0.079) | 4.E-04 | 0.018(0.017) | 0.3 | Light areas cause Cirrus |
|  | Co-twin |  |  | 0.147(0.074) | 5.E-02 | 0.079(0.077) | 0.3 | 0.069(0.029) | 2.E-02 |  |
| Cirrus-light areas | Self | 0.190(0.049) | 9.E-05 |  |  | 0.282(0.079) | 4.E-04 | -0.007(0.008) | 0.4 |  |
|  | Co-twin |  |  | 0.062(0.047) | 0.2 | 0.079(0.077) | 0.3 | -0.018(0.016) | 0.3 |  |
|  |  |  |  |  |  |  |  |  |  |  |
| Bright areas-Cirrus | Self | 0.318(0.080) | 7.E-05 |  |  | 0.298(0.079) | 2.E-04 | 0.020(0.016) | 0.2 | Bright area cause Cirrus |
|  | Co-twin |  |  | 0.219(0.069) | 2.E-03 | 0.183(0.073) | 1.E-02 | 0.036(0.029) | 0.2 |  |
| Cirrus-bright areas | Self | 0.187(0.060) | 2.E-03 |  |  | 0.166(0.057) | 3.E-03 | 0.021(0.014) | 0.1 |  |
|  | Co-twin |  |  | 0.166(0.057) | 3.E-03 | 0.146(0.056) | 1.E-02 | 0.020(0.016) | 0.2 |  |
|  |  |  |  |  |  |  |  |  |  |  |
| Brightest areas (Cirrocumulus)-Cirrus | Self | 0.188(0.085) | 3.E-02 |  |  | 0.198(0.086) | 2.E-02 | -0.010(0.011) | 0.3 | Cirrus causes brightest areas |
|  | Co-twin |  |  | 0.115(0.066) | 8.E-02 | 0.131(0.066) | 5.E-02 | -0.016(0.016) | 0.3 |  |
| Cirrus-brightest areas (Cirrocumulus) | Self | 0.156(0.066) | 2.E-02 |  |  | 0.146(0.065) | 3.E-02 | 0.010(0.009) | 0.2 |  |
|  | Co-twin |  |  | 0.100(0.054) | 6.E-02 | 0.081(0.053) | 0.1 | 0.019(0.013) | 0.1 |  |
|  |  |  |  |  |  |  |  |  |  |  |
| Light areas-brightest areas (Cirrocumulus) | Self | 0.311(0.064) | 1.E-06 |  |  | 0.263(0.071) | 2.E-04 | 0.048(0.023) | 3.E-02 | Light areas cause brightest area |
|  | Co-twin |  |  | 0.235(0.055) | 2.E-05 | 0.144(0.061) | 2.E-02 | 0.091(0.031) | 3.E-03 |  |
| Brightest areas (Cirrocumulus)-light areas | Self | 0.226(0.061) | 2.E-04 |  |  | 0.265(0.059) | 9.E-06 | -0.038(0.017) | 2.E-02 |  |
|  | Co-twin |  |  | 0.137(0.051) | 8.E-03 | 0.192(0.048) | 6.E-05 | -0.055(0.022) | 1.E-02 |  |
|  |  |  |  |  |  |  |  |  |  |  |
| Bright areas- brightest areas (Cirrocumulus) | Self | 0.619(0.046) | 8.E-41 |  |  | 0.593(0.049) | 3.E-34 | 0.026(0.015) | 8.E-02 | Bright areas cause brightest areas |
|  | Co-twin |  |  | 0.363(0.058) | 3.E-10 | 0.084(0.044) | 6.E-02 | 0.278(0.047) | 3.E-09 |  |
| Brightest areas (Cirrocumulus)-bright areas | Self | 0.571(0.051) | 8.E-29 |  |  | 0.556(0.049) | 1.E-29 | 0.015(0.013) | 0.2 |  |
|  | Co-twin |  |  | 0.244(0.062) | 8.E-05 | 0.156(0.044) | 5.E-04 | 0.088(0.042) | 3.E-02 |  |

se standard error

Model 1: Y_self_ = β_self_X_self_ + ε_1_, Model 2: Y_self_ = β_cotwin_X_cotwin_ + ε_2_, Model 3: Y_self_ = β_self_X_self_ + β_cotwin_X_cotwin_ + ε_3_

*change refers to the change in coefficients from Model 1 to Model 3 for woman herself, the change in coefficients from Model 2 to Model 3 for woman’s cotwin; percent mammographic density is calculated based on the threshold of Cumulus measure, 30.5% is the median value.

**Table S4 The relationships between Cirrus and mammographic density measures for percent mammographic density > 30.5% analysed by using the ICE FALCON method**

| **Assignment**  **of X-Y** |  | **Model 1** |  | **Model 2** |  | **Model 3** |  | **Change*** |  | **Causal relationship conclusion** |
| --- | --- | --- | --- | --- | --- | --- | --- | --- | --- | --- |
|  |  | **Coef. (se)** | ***P*** | **Coef. (se)** | ***P*** | **Coef. (se)** | ***P*** | **Coef. (se)** | ***P*** |  |
| Light areas-bright areas | Self | 0.635(0.078) | 4.E-16 |  |  | 0.626(0.077) | 6.E-16 | 0.009(0.014) | 0.5 | Light areas cause bright areas |
|  | Co-twin |  |  | 0.163(0.076) | 3.E-02 | 0.036(0.052) | 0.5 | 0.127(0.051) | 1E-02 |  |
| Bright areas-light areas | Self | 0.683(0.058) | 5.E-32 |  |  | 0.674(0.057) | 5.E-32 | 0.009(0.011) | 0.4 | No clear conclusion |
|  | Co-twin |  |  | 0.094(0.082) | 0.3 | 0.105(0.051) | 4.E-02 | -0.012(0.067) | 0.9 |  |
|  |  |  |  |  |  |  |  |  |  |  |
| Light areas-Cirrus | Self | 0.265(0.092) | 4.E-03 |  |  | 0.254(0.092) | 6.E-03 | 0.011(0.016) | 0.5 | Light areas cause Cirrus |
|  | Co-twin |  |  | 0.105(0.089) | 0.2 | 0.068(0.087) | 0.4 | 0.037(0.024) | 0.1 |  |
| Cirrus-light areas | Self | 0.175(0.059) | 3.E-03 |  |  | 0.171(0.057) | 3.E-03 | 0.004(0.007) | 0.6 |  |
|  | Co-twin |  |  | 0.084(0.059) | 0.2 | 0.081(0.059) | 0.2 | 0.003(0.013) | 0.8 |  |
|  |  |  |  |  |  |  |  |  |  |  |
| Bright areas-Cirrus | Self | 0.260(0.102) | 1.E-02 |  |  | 0.248(0.096) | 1.E-02 | 0.012(0.023) | 0.6 | No clear conclusion |
|  | Co-twin |  |  | 0.237(0.102) | 2.E-02 | 0.231(0.102) | 2.E-02 | 0.006(0.025) | 0.8 |  |
| Cirrus-bright areas | Self | 0.187(0.060) | 2.E-03 |  |  | 0.166(0.057) | 3.E-03 | 0.021(0.014) | 0.1 |  |
|  | Co-twin |  |  | 0.166(0.057) | 3.E-03 | 0.146(0.056) | 1.E-02 | 0.020(0.016) | 0.2 |  |
|  |  |  |  |  |  |  |  |  |  |  |
| Brightest areas (Cirrocumulus)-Cirrus | Self | 0.144(0.092) | 0.1 |  |  | 0.127(0.094) | 0.2 | 0.018(0.017) | 0.3 | No clear conclusion |
|  | Co-twin |  |  | 0.211(0.076) | 6.E-03 | 0.194(0.074) | 9.E-03 | 0.017(0.014) | 0.2 |  |
| Cirrus-brightest areas (Cirrocumulus) | Self | 0.137(0.076) | 7.E-02 |  |  | 0.118(0.071) | 0.1 | 0.019(0.017) | 0.3 |  |
|  | Co-twin |  |  | 0.190(0.064) | 3.E-03 | 0.175(0.063) | 6.E-03 | 0.016(0.015) | 0.3 |  |
|  |  |  |  |  |  |  |  |  |  |  |
| Light areas-brightest areas (Cirrocumulus) | Self | 0.265(0.072) | 3.E-04 |  |  | 0.255(0.072) | 4.E-04 | 0.010(0.016) | 0.5 | Light areas cause brightest areas |
|  | Co-twin |  |  | 0.096(0.076) | 0.2 | 0.055(0.073) | 0.5 | 0.041(0.023) | 7E-02 |  |
| Brightest areas (Cirrocumulus)-light areas | Self | 0.193(0.068) | 5.E-03 |  |  | 0.192(0.070) | 6.E-03 | 0.002(0.006) | 0.8 |  |
|  | Co-twin |  |  | 0.047(0.065) | 0.5 | 0.041(0.063) | 5.E-01 | 0.005(0.017) | 0.8 |  |
|  |  |  |  |  |  |  |  |  |  |  |
| Bright areas- brightest areas (Cirrocumulus) | Self | 0.644(0.060) | 5.E-27 |  |  | 0.630(0.060) | 1.E-25 | 0.014(0.013) | 0.3 | Bright areas cause brightest areas |
|  | Co-twin |  |  | 0.179(0.085) | 4.E-02 | 0.104(0.063) | 0.1 | 0.075(0.062) | 0.2 |  |
| Brightest areas (Cirrocumulus)-bright areas | Self | 0.504(0.051) | 4.E-23 |  |  | 0.495(0.051) | 7.E-22 | 0.009(0.012) | 0.4 |  |
|  | Co-twin |  |  | 0.138(0.069) | 4.E-02 | 0.144(0.092) | 0.1 | -0.006(0.025) | 0.8 |  |

se standard error

Model 1: Y_self_ = β_self_X_self_ + ε_1_, Model 2: Y_self_ = β_cotwin_X_cotwin_ + ε_2_, Model 3: Y_self_ = β_self_X_self_ + β_cotwin_X_cotwin_ + ε_3_

*change refers to the change in coefficients from Model 1 to Model 3 for woman herself, the change in coefficients from Model 2 to Model 3 for woman’s cotwin; percent mammographic density is calculated based on the threshold of Cumulus measure, 30.5% is the median value.
